# Evaluation of a continuous non-invasive arterial blood pressure monitoring device in comparison with an arterial blood pressure measurement in the ICU

**DOI:** 10.1186/cc9492

**Published:** 2011-03-11

**Authors:** K Smolle, M Schmid

**Affiliations:** 1 University Hospital, Graz, Austria; 2 Department of Internal Medicine, Graz, Austria

## Introduction

Due to a lower risk of complications, non-invasive monitoring methods gain importance. Measuring arterial blood pressure belongs to the standard hemodynamic monitoring. A newly developed continuous non-invasive arterial blood pressure (CNAP) measurement method is available and has been validated perioperatively [1]. We compared the CNAP monitoring device with invasive arterial blood pressure measurement (IBP) as the gold standard in critically ill patients.

## Methods

We performed a prospective study on 49 critically ill patients at a medical ICU. All patients were sedated and mechanically ventilated (BIPAP, tidal volume 7 to 8 ml/kg ideal body weight). Furthermore, all patients were under vasopressor therapy. CNAP was applied on two fingers of the hand contralateral to the invasive arterial blood pressure catheter in the A. radialis. All measurements were digitally recorded with a sample frequency of 100 Hz, every pulse beat was automatically identified by an algorithm [2] and subsequently artefacts were removed from the datasets. The average recording time in each patient was 163 minutes (±37 minutes/patient).

## Results

**Figure 1:**
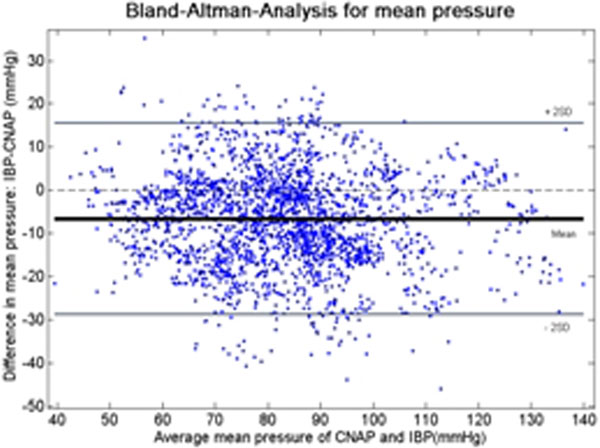
**Comparison between IBP and CNAP in 46 patients (50 beats per patient)**.

In total we analysed 500,000 beats. Overall we observed a bias in mean pressure of -7.49 mmHg with a standard deviation of 10.90 mmHg. The Bland-Altman plot (Figure 1) showed a uniform distribution of the variances over all measured blood pressure values and a good agreement of the mean blood pressure between CNAP and IBP. When analysing the data of each individual patient, larger differences were found. The bias ranged from 0.28 to 23.9 mmHg (median = -6.6 mmHg), with a standard deviation between 2.0 and 14.9 mmHg (median = 5.8 mmHg).

## Conclusions

In our study we detected a good overall agreement between CNAP and IBP. The future perspective of this study is to investigate whether the continuous non-invasive blood pressure waveform is suitable for deriving further hemodynamic parameters of fluid responsiveness.
